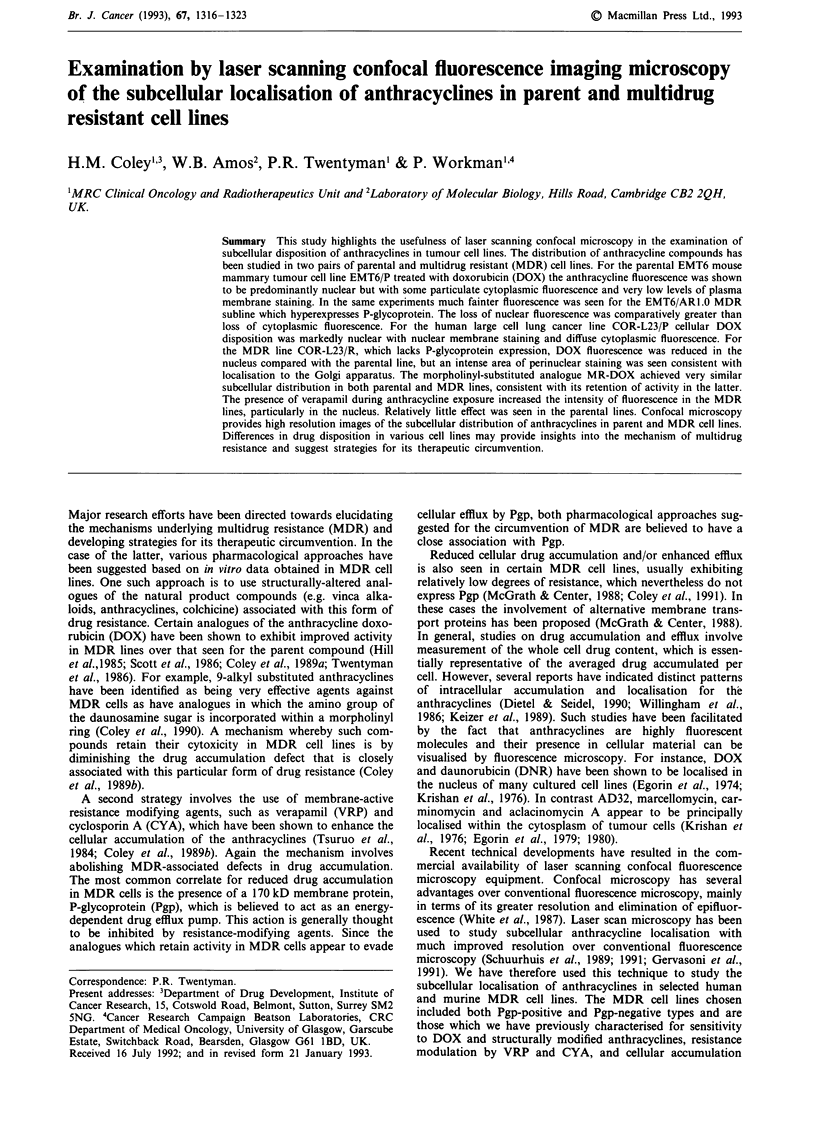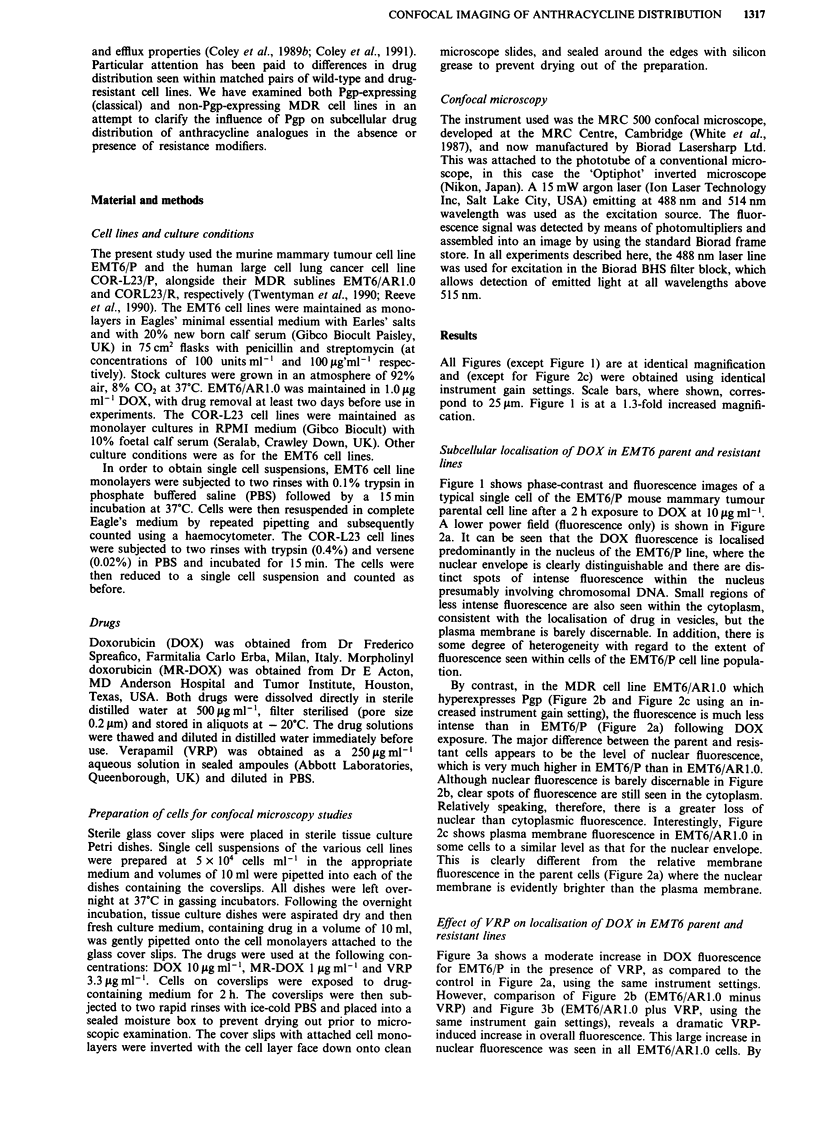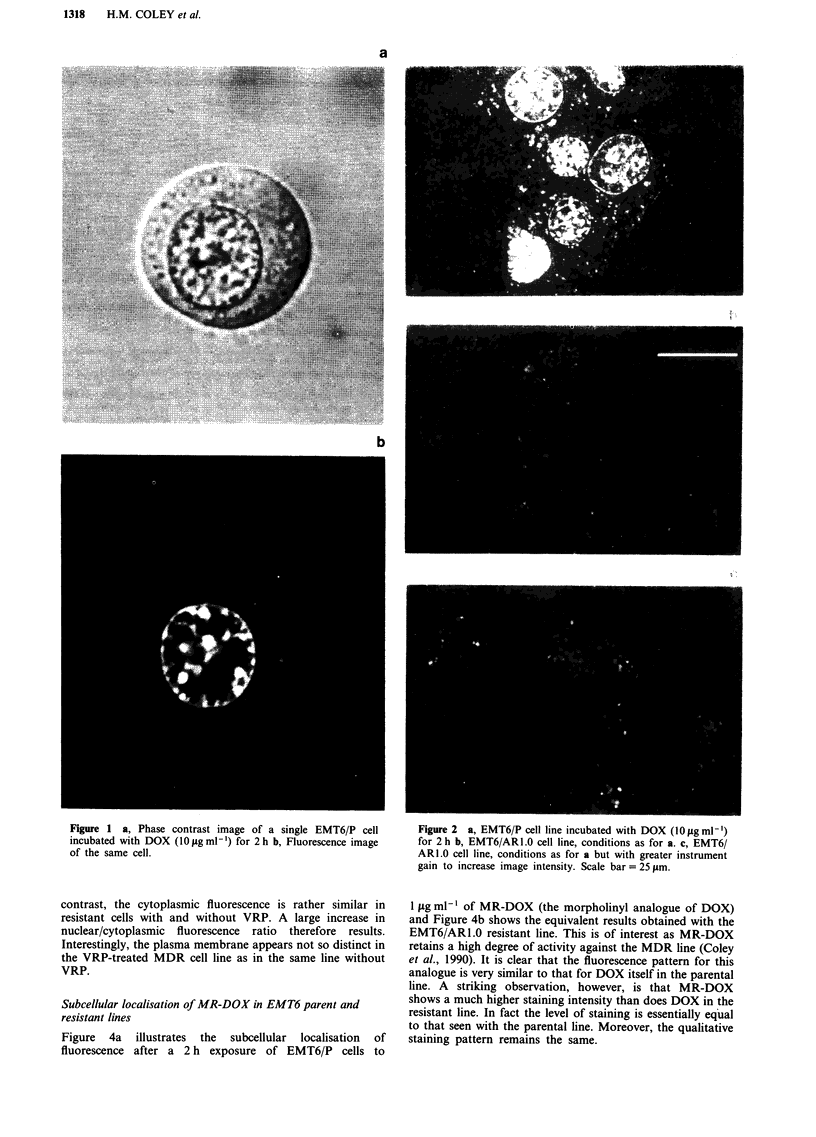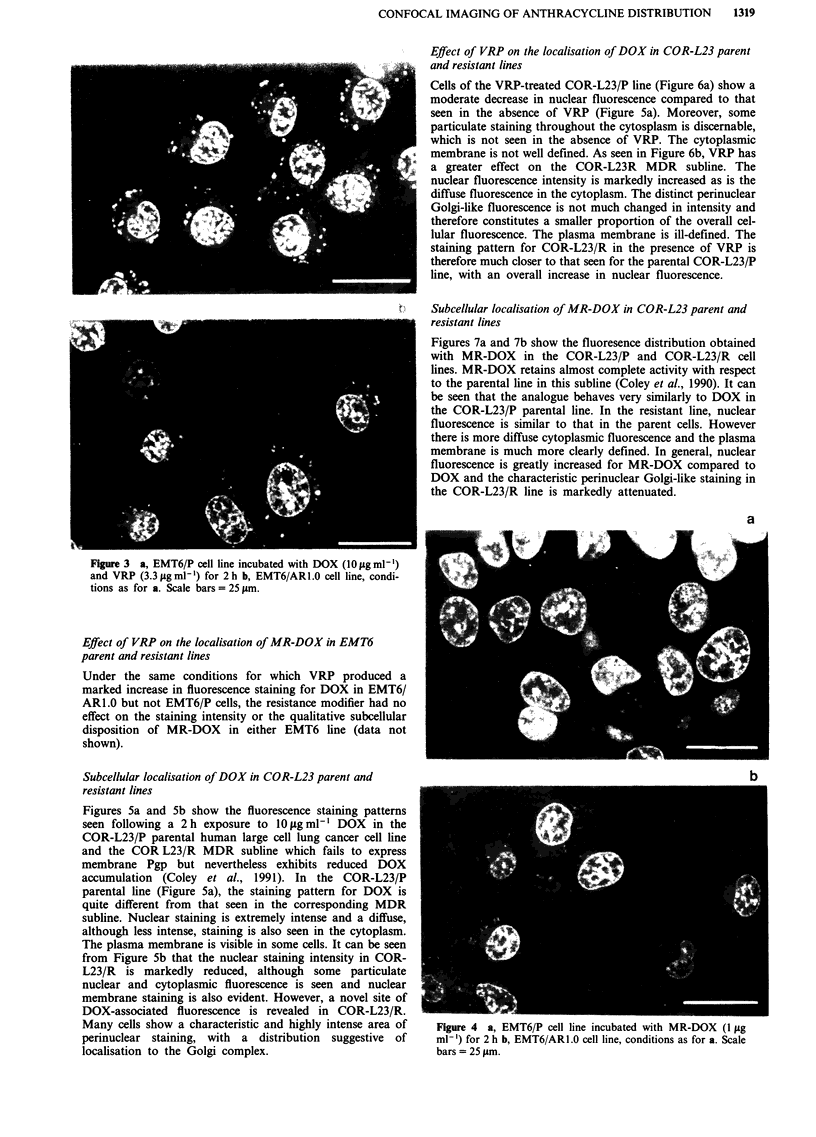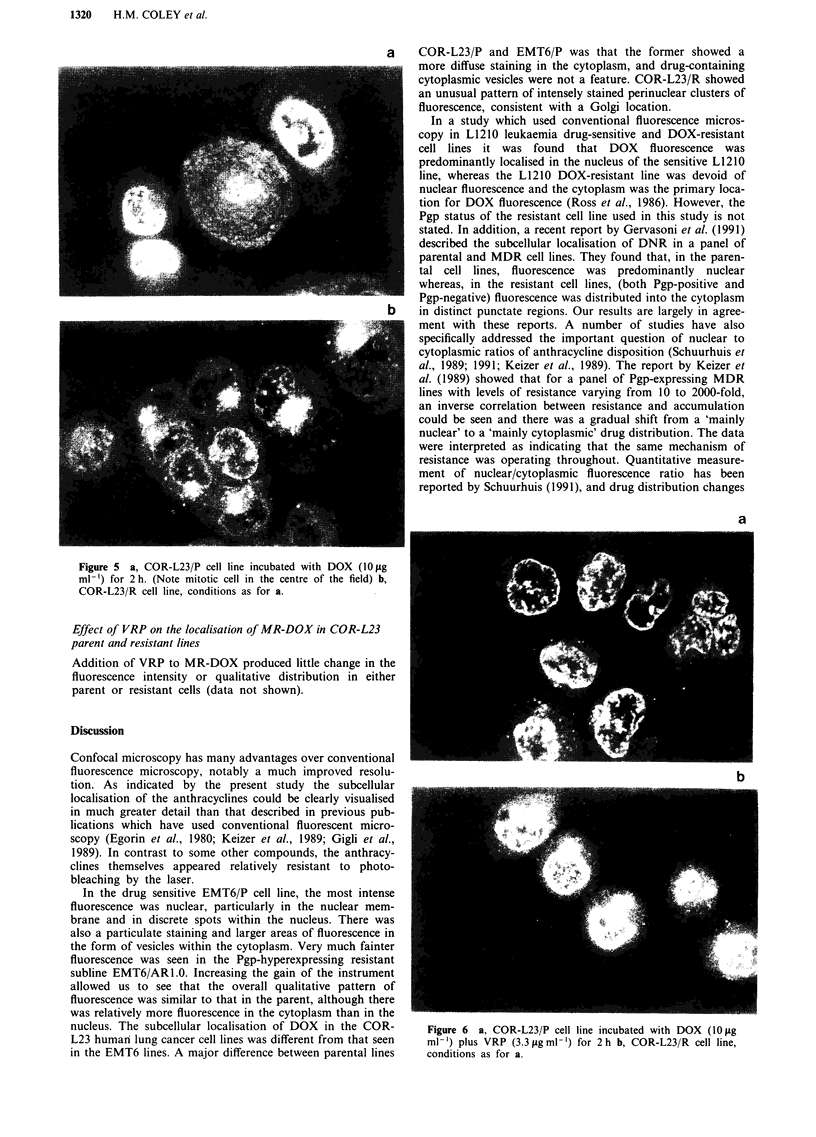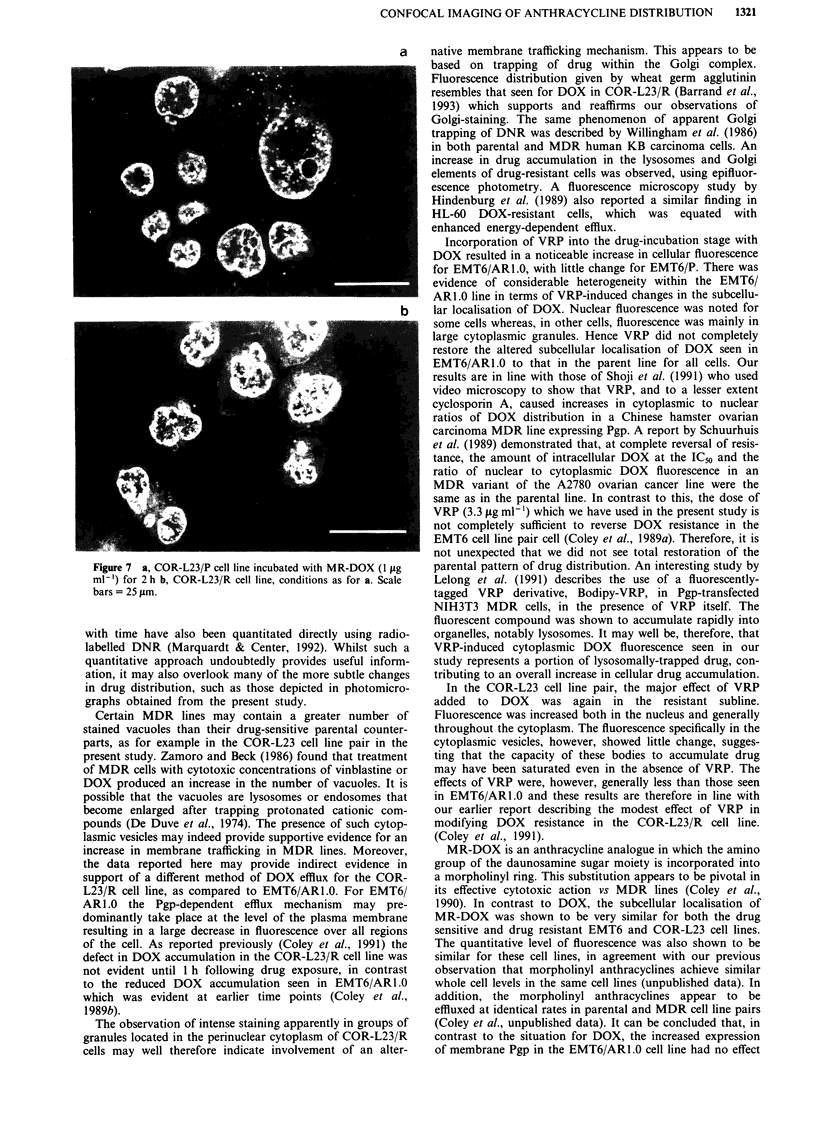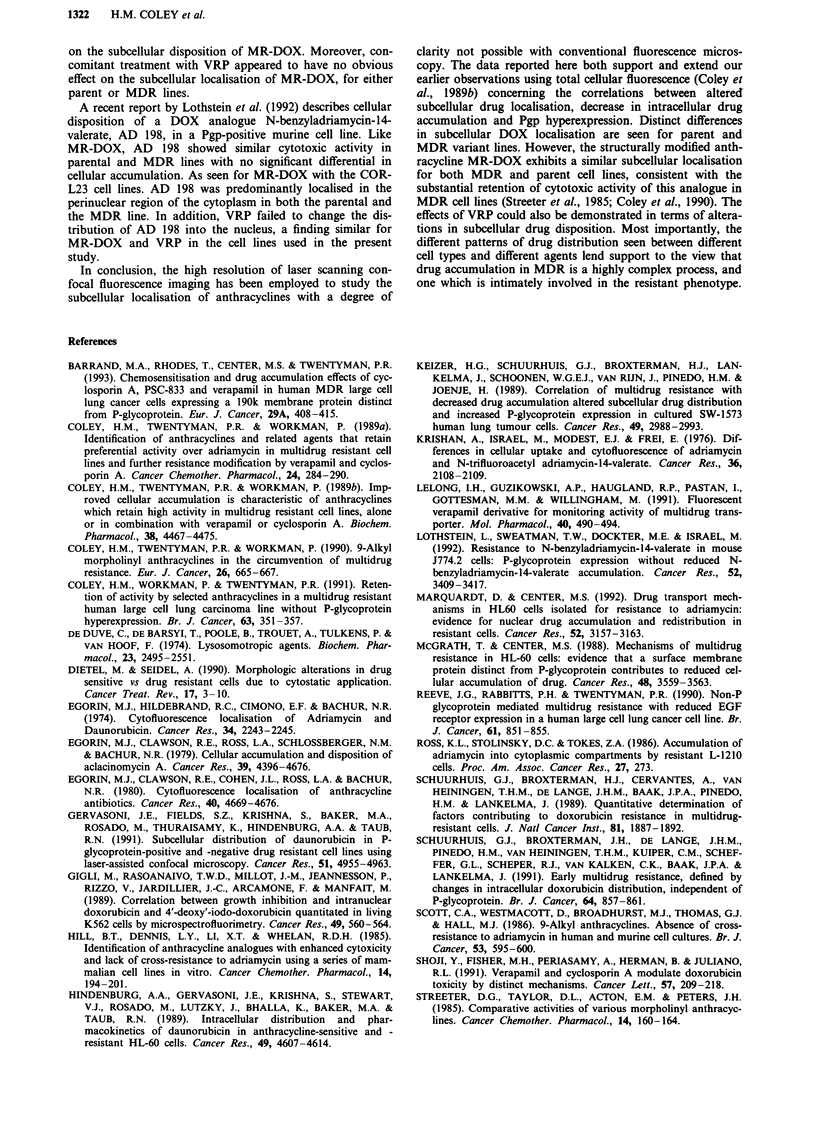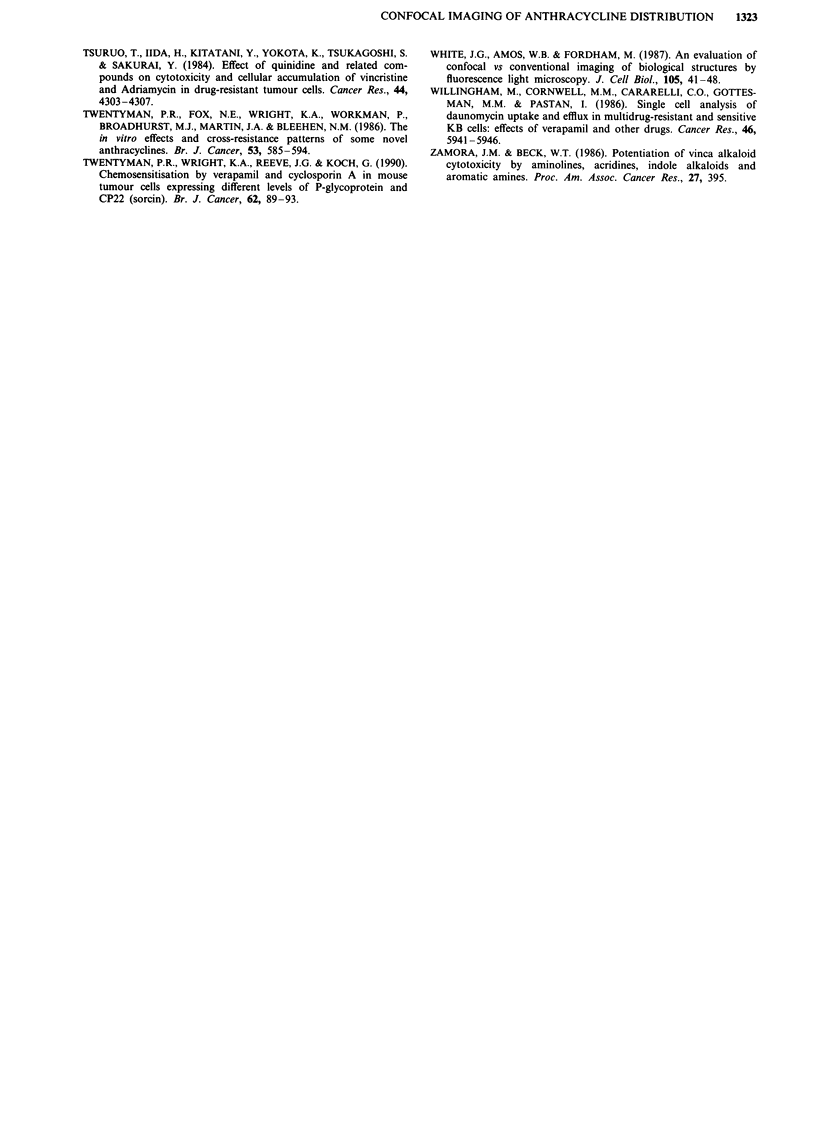# Examination by laser scanning confocal fluorescence imaging microscopy of the subcellular localisation of anthracyclines in parent and multidrug resistant cell lines.

**DOI:** 10.1038/bjc.1993.244

**Published:** 1993-06

**Authors:** H. M. Coley, W. B. Amos, P. R. Twentyman, P. Workman

**Affiliations:** MRC Clinical Oncology and Radiotherapeutics Unit, Cambridge, UK.

## Abstract

**Images:**


### Examination by laser scanning confocal fluorescence imaging microscopy of the subcellular localisation of anthracyclines in parent and multidrug resistant cell lines

H.M. Coley[1,3], W.B. Amos[2], P.R. Twentyman[1] & P. Workman[1,4]

[1]*MRC Clinical Oncology and Radiotherapeutics Unit and* [2]*Laboratory of Molecular Biology, Hills Road, Cambridge CB2 2QH, UK.*

**Summary** This study highlights the usefulness of laser scanning confocal microscopy in the examination of subcellular disposition of anthracyclines in tumour cell lines. The distribution of anthracycline compounds has been studied in two pairs of parental and multidrug resistant (MDR) cell lines. For the parental EMT6 mouse mammary tumour cell line EMT6/P treated with doxorubicin (DOX) the anthracycline fluorescence was shown to be predominantly nuclear but with some particulate cytoplasmic fluorescence and very low levels of plasma membrane staining. In the same experiments much fainter fluorescence was seen for the EMT6/AR1.0 MDR subline which hyperexpresses P-glycoprotein. The loss of nuclear fluorescence was comparatively greater than loss of cytoplasmic fluorescence. For the human large cell lung cancer line COR-L23/P cellular DOX disposition was markedly nuclear with nuclear membrane staining and diffuse cytoplasmic fluorescence. For the MDR line COR-L23/R, which lacks P-glycoprotein expression, DOX fluorescence was reduced in the nucleus compared with the parental line, but an intense area of perinuclear staining was seen consistent with localisation to the Golgi apparatus. The morpholinyl-substituted analogue MR-DOX achieved very similar subcellular distribution in both parental and MDR lines, consistent with its retention of activity in the latter. The presence of verapamil during anthracycline exposure increased the intensity of fluorescence in the MDR lines, particularly in the nucleus. Relatively little effect was seen in the parental lines. Confocal microscopy provides high resolution images of the subcellular distribution of anthracyclines in parent and MDR cell lines. Differences in drug disposition in various cell lines may provide insights into the mechanism of multidrug resistance and suggest strategies for its therapeutic circumvention.

Major research efforts have been directed towards elucidating the mechanisms underlying multidrug resistance (MDR) and developing strategies for its therapeutic circumvention. In the case of the latter, various pharmacological approaches have been suggested based on *in vitro* data obtained in MDR cell lines. One such approach is to use structurally-altered analogues of the natural product compounds (e.g. vinca alkaloids, anthracyclines, colchicine) associated with this form of drug resistance. Certain analogues of the anthracycline doxorubicin (DOX) have been shown to exhibit improved activity in MDR lines over that seen for the parent compound (Hill *et al.*,1985; Scott *et al.*, 1986; Coley *et al.*, 1989*a*; Twentyman *et al.*, 1986). For example, 9-alkyl substituted anthracyclines have been identified as being very effective agents against MDR cells as have analogues in which the amino group of the daunosamine sugar is incorporated within a morpholinyl ring (Coley *et al.*, 1990). A mechanism whereby such compounds retain their cytoxicity in MDR cell lines is by diminishing the drug accumulation defect that is closely associated with this particular form of drug resistance (Coley *et al.*, 1989*b*).

A second strategy involves the use of membrane-active resistance modifying agents, such as verapamil (VRP) and cyclosporin A (CYA), which have been shown to enhance the cellular accumulation of the anthracyclines (Tsuruo *et al.*, 1984; Coley *et al.*, 1989*b*). Again the mechanism involves abolishing MDR-associated defects in drug accumulation. The most common correlate for reduced drug accumulation in MDR cells is the presence of a 170 kD membrane protein, P-glycoprotein (Pgp), which is believed to act as an energy-dependent drug efflux pump. This action is generally thought to be inhibited by resistance-modifying agents. Since the analogues which retain activity in MDR cells appear to evade cellular efflux by Pgp, both pharmacological approaches suggested for the circumvention of MDR are believed to have a close association with Pgp.

Reduced cellular drug accumulation and/or enhanced efflux is also seen in certain MDR cell lines, usually exhibiting relatively low degrees of resistance, which nevertheless do not express Pgp (McGrath & Center, 1988; Coley *et al.*, 1991). In these cases the involvement of alternative membrane transport proteins has been proposed (McGrath & Center, 1988). In general, studies on drug accumulation and efflux involve measurement of the whole cell drug content, which is essentially representative of the averaged drug accumulated per cell. However, several reports have indicated distinct patterns of intracellular accumulation and localisation for the anthracyclines (Dietel & Seidel, 1990; Willingham *et al.*, 1986; Keizer *et al.*, 1989). Such studies have been facilitated by the fact that anthracyclines are highly fluorescent molecules and their presence in cellular material can be visualised by fluorescence microscopy. For instance, DOX and daunorubicin (DNR) have been shown to be localised in the nucleus of many cultured cell lines (Egorin *et al.*, 1974; Krishan *et al.*, 1976). In contrast AD32, marcellomycin, carminomycin and aclacinomycin A appear to be principally localised within the cytosplasm of tumour cells (Krishan *et al.*, 1976; Egorin *et al.*, 1979; 1980).

Recent technical developments have resulted in the commercial availability of laser scanning confocal fluorescence microscopy equipment. Confocal microscopy has several advantages over conventional fluorescence microscopy, mainly in terms of its greater resolution and elimination of epifluorescence (White *et al.*, 1987). Laser scan microscopy has been used to study subcellular anthracycline localisation with much improved resolution over conventional fluorescence microscopy (Schuurhuis *et al.*, 1989; 1991; Gervasoni *et al.*, 1991). We have therefore used this technique to study the subcellular localisation of anthracyclines in selected human and murine MDR cell lines. The MDR cell lines chosen included both Pgp-positive and Pgp-negative types and are those which we have previously characterised for sensitivity to DOX and structurally modified anthracyclines, resistance modulation by VRP and CYA, and cellular accumulation

Correspondence: P.R. Twentyman.
Present addresses: [3]Department of Drug Development, Institute of Cancer Research, 15, Cotswold Road, Belmont, Sutton, Surrey SM2 5NG. [4]Cancer Research Campaign Beatson Laboratories, CRC Department of Medical Oncology, University of Glasgow, Garscube Estate, Switchback Road, Bearsden, Glasgow G61 1BD, UK.
Received 16 July 1992; and in revised form 21 January 1993.

and efflux properties (Coley *et al.*, 1989*b*; Coley *et al.*, 1991). Particular attention has been paid to differences in drug distribution seen within matched pairs of wild-type and drug-resistant cell lines. We have examined both Pgp-expressing (classical) and non-Pgp-expressing MDR cell lines in an attempt to clarify the influence of Pgp on subcellular drug distribution of anthracycline analogues in the absence or presence of resistance modifiers.

### Material and methods

### *Cell lines and culture conditions*

The present study used the murine mammary tumour cell line EMT6/P and the human large cell lung cancer cell line COR-L23/P, alongside their MDR sublines EMT6/AR1.0 and CORL23/R, respectively (Twentyman *et al.*, 1990; Reeve *et al.*, 1990). The EMT6 cell lines were maintained as monolayers in Eagles' minimal essential medium with Earles' salts and with 20% new born calf serum (Gibco Biocult Paisley, UK) in 75 $cm^2$ flasks with penicillin and streptomycin (at concentrations of 100 units $ml^{-1}$ and 100 $\mu g' ml^{-1}$ respectively). Stock cultures were grown in an atmosphere of 92% air, 8% $CO_2$ at 37°C. EMT6/AR1.0 was maintained in 1.0 $\mu g$ $ml^{-1}$ DOX, with drug removal at least two days before use in experiments. The COR-L23 cell lines were maintained as monolayer cultures in RPMI medium (Gibco Biocult) with 10% foetal calf serum (Seralab, Crawley Down, UK). Other culture conditions were as for the EMT6 cell lines.

In order to obtain single cell suspensions, EMT6 cell line monolayers were subjected to two rinses with 0.1% trypsin in phosphate buffered saline (PBS) followed by a 15 min incubation at 37°C. Cells were then resuspended in complete Eagle's medium by repeated pipetting and subsequently counted using a haemocytometer. The COR-L23 cell lines were subjected to two rinses with trypsin (0.4%) and versene (0.02%) in PBS and incubated for 15 min. The cells were then reduced to a single cell suspension and counted as before.

### *Drugs*

Doxorubicin (DOX) was obtained from Dr Frederico Spreafico, Farmitalia Carlo Erba, Milan, Italy. Morpholinyl doxorubicin (MR-DOX) was obtained from Dr E Acton, MD Anderson Hospital and Tumor Institute, Houston, Texas, USA. Both drugs were dissolved directly in sterile distilled water at 500 $\mu g\, ml^{-1}$, filter sterilised (pore size 0.2 $\mu m$) and stored in aliquots at − 20°C. The drug solutions were thawed and diluted in distilled water immediately before use. Verapamil (VRP) was obtained as a 250 $\mu g\, ml^{-1}$ aqueous solution in sealed ampoules (Abbott Laboratories, Queenborough, UK) and diluted in PBS.

### *Preparation of cells for confocal microscopy studies*

Sterile glass cover slips were placed in sterile tissue culture Petri dishes. Single cell suspensions of the various cell lines were prepared at $5 \times 10^4$ cells $ml^{-1}$ in the appropriate medium and volumes of 10 ml were pipetted into each of the dishes containing the coverslips. All dishes were left overnight at 37°C in gassing incubators. Following the overnight incubation, tissue culture dishes were aspirated dry and then fresh culture medium, containing drug in a volume of 10 ml, was gently pipetted onto the cell monolayers attached to the glass cover slips. The drugs were used at the following concentrations: DOX 10 $\mu g\, ml^{-1}$, MR-DOX 1 $\mu g\, ml^{-1}$ and VRP 3.3 $\mu g\, ml^{-1}$. Cells on coverslips were exposed to drug-containing medium for 2 h. The coverslips were then subjected to two rapid rinses with ice-cold PBS and placed into a sealed moisture box to prevent drying out prior to microscopic examination. The cover slips with attached cell monolayers were inverted with the cell layer face down onto clean microscope slides, and sealed around the edges with silicon grease to prevent drying out of the preparation.

### *Confocal microscopy*

The instrument used was the MRC 500 confocal microscope, developed at the MRC Centre, Cambridge (White *et al.*, 1987), and now manufactured by Biorad Lasersharp Ltd. This was attached to the phototube of a conventional microscope, in this case the 'Optiphot' inverted microscope (Nikon, Japan). A 15 mW argon laser (Ion Laser Technology Inc, Salt Lake City, USA) emitting at 488 nm and 514 nm wavelength was used as the excitation source. The fluorescence signal was detected by means of photomultipliers and assembled into an image by using the standard Biorad frame store. In all experiments described here, the 488 nm laser line was used for excitation in the Biorad BHS filter block, which allows detection of emitted light at all wavelengths above 515 nm.

### Results

All Figures (except Figure 1) are at identical magnification and (except for Figure 2c) were obtained using identical instrument gain settings. Scale bars, where shown, correspond to 25 $\mu m$. Figure 1 is at a 1.3-fold increased magnification.

### *Subcellular localisation of DOX in EMT6 parent and resistant lines*

Figure 1 shows phase-contrast and fluorescence images of a typical single cell of the EMT6/P mouse mammary tumour parental cell line after a 2 h exposure to DOX at 10 $\mu g\, ml^{-1}$. A lower power field (fluorescence only) is shown in Figure 2a. It can be seen that the DOX fluorescence is localised predominantly in the nucleus of the EMT6/P line, where the nuclear envelope is clearly distinguishable and there are distinct spots of intense fluorescence within the nucleus presumably involving chromosomal DNA. Small regions of less intense fluorescence are also seen within the cytoplasm, consistent with the localisation of drug in vesicles, but the plasma membrane is barely discernable. In addition, there is some degree of heterogeneity with regard to the extent of fluorescence seen within cells of the EMT6/P cell line population.

By contrast, in the MDR cell line EMT6/AR1.0 which hyperexpresses Pgp (Figure 2b and Figure 2c using an increased instrument gain setting), the fluorescence is much less intense than in EMT6/P (Figure 2a) following DOX exposure. The major difference between the parent and resistant cells appears to be the level of nuclear fluorescence, which is very much higher in EMT6/P than in EMT6/AR1.0. Although nuclear fluorescence is barely discernable in Figure 2b, clear spots of fluorescence are still seen in the cytoplasm. Relatively speaking, therefore, there is a greater loss of nuclear than cytoplasmic fluorescence. Interestingly, Figure 2c shows plasma membrane fluorescence in EMT6/AR1.0 in some cells to a similar level as that for the nuclear envelope. This is clearly different from the relative membrane fluorescence in the parent cells (Figure 2a) where the nuclear membrane is evidently brighter than the plasma membrane.

### *Effect of VRP on localisation of DOX in EMT6 parent and resistant lines*

Figure 3a shows a moderate increase in DOX fluorescence for EMT6/P in the presence of VRP, as compared to the control in Figure 2a, using the same instrument settings. However, comparison of Figure 2b (EMT6/AR1.0 minus VRP) and Figure 3b (EMT6/AR1.0 plus VRP, using the same instrument gain settings), reveals a dramatic VRP-induced increase in overall fluorescence. This large increase in nuclear fluorescence was seen in all EMT6/AR1.0 cells. By

**Figure 1** **a**, Phase contrast image of a single EMT6/P cell incubated with DOX ($10\,\mu g\,ml^{-1}$) for 2 h **b**, Fluorescence image of the same cell.

**Figure 2** **a**, EMT6/P cell line incubated with DOX ($10\,\mu g\,ml^{-1}$) for 2 h **b**, EMT6/AR1.0 cell line, conditions as for **a**. **c**, EMT6/AR1.0 cell line, conditions as for **a** but with greater instrument gain to increase image intensity. Scale bar = $25\,\mu m$.

contrast, the cytoplasmic fluorescence is rather similar in resistant cells with and without VRP. A large increase in nuclear/cytoplasmic fluorescence ratio therefore results. Interestingly, the plasma membrane appears not so distinct in the VRP-treated MDR cell line as in the same line without VRP.

*Subcellular localisation of MR-DOX in EMT6 parent and resistant lines*

Figure 4a illustrates the subcellular localisation of fluorescence after a 2 h exposure of EMT6/P cells to $1\,\mu g\,ml^{-1}$ of MR-DOX (the morpholinyl analogue of DOX) and Figure 4b shows the equivalent results obtained with the EMT6/AR1.0 resistant line. This is of interest as MR-DOX retains a high degree of activity against the MDR line (Coley *et al.*, 1990). It is clear that the fluorescence pattern for this analogue is very similar to that for DOX itself in the parental line. A striking observation, however, is that MR-DOX shows a much higher staining intensity than does DOX in the resistant line. In fact the level of staining is essentially equal to that seen with the parental line. Moreover, the qualitative staining pattern remains the same.

**Figure 3** a, EMT6/P cell line incubated with DOX (10 μg ml$^{-1}$) and VRP (3.3 μg ml$^{-1}$) for 2 h b, EMT6/AR1.0 cell line, conditions as for a. Scale bars = 25 μm.

*Effect of VRP on the localisation of MR-DOX in EMT6 parent and resistant lines*

Under the same conditions for which VRP produced a marked increase in fluorescence staining for DOX in EMT6/AR1.0 but not EMT6/P cells, the resistance modifier had no effect on the staining intensity or the qualitative subcellular disposition of MR-DOX in either EMT6 line (data not shown).

*Subcellular localisation of DOX in COR-L23 parent and resistant lines*

Figures 5a and 5b show the fluorescence staining patterns seen following a 2 h exposure to 10 μg ml$^{-1}$ DOX in the COR-L23/P parental human large cell lung cancer cell line and the COR L23/R MDR subline which fails to express membrane Pgp but nevertheless exhibits reduced DOX accumulation (Coley *et al.*, 1991). In the COR-L23/P parental line (Figure 5a), the staining pattern for DOX is quite different from that seen in the corresponding MDR subline. Nuclear staining is extremely intense and a diffuse, although less intense, staining is also seen in the cytoplasm. The plasma membrane is visible in some cells. It can be seen from Figure 5b that the nuclear staining intensity in COR-L23/R is markedly reduced, although some particulate nuclear and cytoplasmic fluorescence is seen and nuclear membrane staining is also evident. However, a novel site of DOX-associated fluorescence is revealed in COR-L23/R. Many cells show a characteristic and highly intense area of perinuclear staining, with a distribution suggestive of localisation to the Golgi complex.

*Effect of VRP on the localisation of DOX in COR-L23 parent and resistant lines*

Cells of the VRP-treated COR-L23/P line (Figure 6a) show a moderate decrease in nuclear fluorescence compared to that seen in the absence of VRP (Figure 5a). Moreover, some particulate staining throughout the cytosplasm is discernable, which is not seen in the absence of VRP. The cytoplasmic membrane is not well defined. As seen in Figure 6b, VRP has a greater effect on the COR-L23R MDR subline. The nuclear fluorescence intensity is markedly increased as is the diffuse fluorescence in the cytoplasm. The distinct perinuclear Golgi-like fluorescence is not much changed in intensity and therefore constitutes a smaller proportion of the overall cellular fluorescence. The plasma membrane is ill-defined. The staining pattern for COR-L23/R in the presence of VRP is therefore much closer to that seen for the parental COR-L23/P line, with an overall increase in nuclear fluorescence.

*Subcellular localisation of MR-DOX in COR-L23 parent and resistant lines*

Figures 7a and 7b show the fluoresence distribution obtained with MR-DOX in the COR-L23/P and COR-L23/R cell lines. MR-DOX retains almost complete activity with respect to the parental line in this subline (Coley *et al.*, 1990). It can be seen that the analogue behaves very similarly to DOX in the COR-L23/P parental line. In the resistant line, nuclear fluorescence is similar to that in the parent cells. However there is more diffuse cytoplasmic fluorescence and the plasma membrane is much more clearly defined. In general, nuclear fluorescence is greatly increased for MR-DOX compared to DOX and the characteristic perinuclear Golgi-like staining in the COR-L23/R line is markedly attenuated.

**Figure 4** a, EMT6/P cell line incubated with MR-DOX (1 μg ml$^{-1}$) for 2 h b, EMT6/AR1.0 cell line, conditions as for a. Scale bars = 25 μm.

**Figure 5** **a**, COR-L23/P cell line incubated with DOX (10 μg $ml^{-1}$) for 2 h. (Note mitotic cell in the centre of the field) **b**, COR-L23/R cell line, conditions as for **a**.

*Effect of VRP on the localisation of MR-DOX in COR-L23 parent and resistant lines*

Addition of VRP to MR-DOX produced little change in the fluorescence intensity or qualitative distribution in either parent or resistant cells (data not shown).

### Discussion

Confocal microscopy has many advantages over conventional fluorescence microscopy, notably a much improved resolution. As indicated by the present study the subcellular localisation of the anthracyclines could be clearly visualised in much greater detail than that described in previous publications which have used conventional fluorescent microscopy (Egorin *et al*., 1980; Keizer *et al*., 1989; Gigli *et al*., 1989). In contrast to some other compounds, the anthracyclines themselves appeared relatively resistant to photobleaching by the laser.

In the drug sensitive EMT6/P cell line, the most intense fluorescence was nuclear, particularly in the nuclear membrane and in discrete spots within the nucleus. There was also a particulate staining and larger areas of fluorescence in the form of vesicles within the cytoplasm. Very much fainter fluorescence was seen in the Pgp-hyperexpressing resistant subline EMT6/AR1.0. Increasing the gain of the instrument allowed us to see that the overall qualitative pattern of fluorescence was similar to that in the parent, although there was relatively more fluorescence in the cytoplasm than in the nucleus. The subcellular localisation of DOX in the COR-L23 human lung cancer cell lines was different from that seen in the EMT6 lines. A major difference between parental lines COR-L23/P and EMT6/P was that the former showed a more diffuse staining in the cytoplasm, and drug-containing cytoplasmic vesicles were not a feature. COR-L23/R showed an unusual pattern of intensely stained perinuclear clusters of fluorescence, consistent with a Golgi location.

In a study which used conventional fluorescence microscopy in L1210 leukaemia drug-sensitive and DOX-resistant cell lines it was found that DOX fluorescence was predominantly localised in the nucleus of the sensitive L1210 line, whereas the L1210 DOX-resistant line was devoid of nuclear fluorescence and the cytoplasm was the primary location for DOX fluorescence (Ross *et al*., 1986). However, the Pgp status of the resistant cell line used in this study is not stated. In addition, a recent report by Gervasoni *et al*. (1991) described the subcellular localisation of DNR in a panel of parental and MDR cell lines. They found that, in the parental cell lines, fluorescence was predominantly nuclear whereas, in the resistant cell lines, (both Pgp-positive and Pgp-negative) fluorescence was distributed into the cytoplasm in distinct punctate regions. Our results are largely in agreement with these reports. A number of studies have also specifically addressed the important question of nuclear to cytoplasmic ratios of anthracycline disposition (Schuurhuis *et al*., 1989; 1991; Keizer *et al*., 1989). The report by Keizer *et al*. (1989) showed that for a panel of Pgp-expressing MDR lines with levels of resistance varying from 10 to 2000-fold, an inverse correlation between resistance and accumulation could be seen and there was a gradual shift from a 'mainly nuclear' to a 'mainly cytoplasmic' drug distribution. The data were interpreted as indicating that the same mechanism of resistance was operating throughout. Quantitative measurement of nuclear/cytoplasmic fluorescence ratio has been reported by Schuurhuis (1991), and drug distribution changes

**Figure 6** **a**, COR-L23/P cell line incubated with DOX (10 μg $ml^{-1}$) plus VRP (3.3 μg $ml^{-1}$) for 2 h **b**, COR-L23/R cell line, conditions as for **a**.

**Figure 7** **a**, COR-L23/P cell line incubated with MR-DOX (1 μg $ml^{-1}$) for 2 h **b**, COR-L23/R cell line, conditions as for **a**. Scale bars = 25 μm.

with time have also been quantitated directly using radiolabelled DNR (Marquardt & Center, 1992). Whilst such a quantitative approach undoubtedly provides useful information, it may also overlook many of the more subtle changes in drug distribution, such as those depicted in photomicrographs obtained from the present study.

Certain MDR lines may contain a greater number of stained vacuoles than their drug-sensitive parental counterparts, as for example in the COR-L23 cell line pair in the present study. Zamoro and Beck (1986) found that treatment of MDR cells with cytotoxic concentrations of vinblastine or DOX produced an increase in the number of vacuoles. It is possible that the vacuoles are lysosomes or endosomes that become enlarged after trapping protonated cationic compounds (De Duve *et al.*, 1974). The presence of such cytoplasmic vesicles may indeed provide supportive evidence for an increase in membrane trafficking in MDR lines. Moreover, the data reported here may provide indirect evidence in support of a different method of DOX efflux for the COR-L23/R cell line, as compared to EMT6/AR1.0. For EMT6/AR1.0 the Pgp-dependent efflux mechanism may predominantly take place at the level of the plasma membrane resulting in a large decrease in fluorescence over all regions of the cell. As reported previously (Coley *et al.*, 1991) the defect in DOX accumulation in the COR-L23/R cell line was not evident until 1 h following drug exposure, in contrast to the reduced DOX accumulation seen in EMT6/AR1.0 which was evident at earlier time points (Coley *et al.*, 1989*b*).

The observation of intense staining apparently in groups of granules located in the perinuclear cytoplasm of COR-L23/R cells may well therefore indicate involvement of an alternative membrane trafficking mechanism. This appears to be based on trapping of drug within the Golgi complex. Fluorescence distribution given by wheat germ agglutinin resembles that seen for DOX in COR-L23/R (Barrand *et al.*, 1993) which supports and reaffirms our observations of Golgi-staining. The same phenomenon of apparent Golgi trapping of DNR was described by Willingham *et al.* (1986) in both parental and MDR human KB carcinoma cells. An increase in drug accumulation in the lysosomes and Golgi elements of drug-resistant cells was observed, using epifluorescence photometry. A fluorescence microscopy study by Hindenburg *et al.* (1989) also reported a similar finding in HL-60 DOX-resistant cells, which was equated with enhanced energy-dependent efflux.

Incorporation of VRP into the drug-incubation stage with DOX resulted in a noticeable increase in cellular fluorescence for EMT6/AR1.0, with little change for EMT6/P. There was evidence of considerable heterogeneity within the EMT6/AR1.0 line in terms of VRP-induced changes in the subcellular localisation of DOX. Nuclear fluorescence was noted for some cells whereas, in other cells, fluorescence was mainly in large cytoplasmic granules. Hence VRP did not completely restore the altered subcellular localisation of DOX seen in EMT6/AR1.0 to that in the parent line for all cells. Our results are in line with those of Shoji *et al.* (1991) who used video microscopy to show that VRP, and to a lesser extent cyclosporin A, caused increases in cytoplasmic to nuclear ratios of DOX distribution in a Chinese hamster ovarian carcinoma MDR line expressing Pgp. A report by Schuurhuis *et al.* (1989) demonstrated that, at complete reversal of resistance, the amount of intracellular DOX at the $IC_{50}$ and the ratio of nuclear to cytoplasmic DOX fluorescence in an MDR variant of the A2780 ovarian cancer line were the same as in the parental line. In contrast to this, the dose of VRP (3.3 μg $ml^{-1}$) which we have used in the present study is not completely sufficient to reverse DOX resistance in the EMT6 cell line pair cell (Coley *et al.*, 1989*a*). Therefore, it is not unexpected that we did not see total restoration of the parental pattern of drug distribution. An interesting study by Lelong *et al.* (1991) describes the use of a fluorescently-tagged VRP derivative, Bodipy-VRP, in Pgp-transfected NIH3T3 MDR cells, in the presence of VRP itself. The fluorescent compound was shown to accumulate rapidly into organelles, notably lysosomes. It may well be, therefore, that VRP-induced cytoplasmic DOX fluorescence seen in our study represents a portion of lysosomally-trapped drug, contributing to an overall increase in cellular drug accumulation.

In the COR-L23 cell line pair, the major effect of VRP added to DOX was again in the resistant subline. Fluorescence was increased both in the nucleus and generally throughout the cytoplasm. The fluorescence specifically in the cytoplasmic vesicles, however, showed little change, suggesting that the capacity of these bodies to accumulate drug may have been saturated even in the absence of VRP. The effects of VRP were, however, generally less than those seen in EMT6/AR1.0 and these results are therefore in line with our earlier report describing the modest effect of VRP in modifying DOX resistance in the COR-L23/R cell line. (Coley *et al.*, 1991).

MR-DOX is an anthracycline analogue in which the amino group of the daunosamine sugar moiety is incorporated into a morpholinyl ring. This substitution appears to be pivotal in its effective cytotoxic action *vs* MDR lines (Coley *et al.*, 1990). In contrast to DOX, the subcellular localisation of MR-DOX was shown to be very similar for both the drug sensitive and drug resistant EMT6 and COR-L23 cell lines. The quantitative level of fluorescence was also shown to be similar for these cell lines, in agreement with our previous observation that morpholinyl anthracyclines achieve similar whole cell levels in the same cell lines (unpublished data). In addition, the morpholinyl anthracyclines appear to be effluxed at identical rates in parental and MDR cell line pairs (Coley *et al.*, unpublished data). It can be concluded that, in contrast to the situation for DOX, the increased expression of membrane Pgp in the EMT6/AR1.0 cell line had no effect on the subcellular disposition of MR-DOX. Moreover, concomitant treatment with VRP appeared to have no obvious effect on the subcellular localisation of MR-DOX, for either parent or MDR lines.

A recent report by Lothstein et al. (1992) describes cellular disposition of a DOX analogue N-benzyladriamycin-14-valerate, AD 198, in a Pgp-positive murine cell line. Like MR-DOX, AD 198 showed similar cytotoxic activity in parental and MDR lines with no significant differential in cellular accumulation. As seen for MR-DOX with the COR-L23 cell lines. AD 198 was predominantly localised in the perinuclear region of the cytoplasm in both the parental and the MDR line. In addition, VRP failed to change the distribution of AD 198 into the nucleus, a finding similar for MR-DOX and VRP in the cell lines used in the present study.

In conclusion, the high resolution of laser scanning confocal fluorescence imaging has been employed to study the subcellular localisation of anthracyclines with a degree of clarity not possible with conventional fluorescence microscopy. The data reported here both support and extend our earlier observations using total cellular fluorescence (Coley et al., 1989b) concerning the correlations between altered subcellular drug localisation, decrease in intracellular drug accumulation and Pgp hyperexpression. Distinct differences in subcellular DOX localisation are seen for parent and MDR variant lines. However, the structurally modified anthracycline MR-DOX exhibits a similar subcellular localisation for both MDR and parent cell lines, consistent with the substantial retention of cytotoxic activity of this analogue in MDR cell lines (Streeter et al., 1985; Coley et al., 1990). The effects of VRP could also be demonstrated in terms of alterations in subcellular drug disposition. Most importantly, the different patterns of drug distribution seen between different cell types and different agents lend support to the view that drug accumulation in MDR is a highly complex process, and one which is intimately involved in the resistant phenotype.

### References

BARRAND, M.A., RHODES, T., CENTER, M.S. & TWENTYMAN, P.R. (1993). Chemosensitisation and drug accumulation effects of cyclosporin A, PSC-833 and verapamil in human MDR large cell lung cancer cells expressing a 190k membrane protein distinct from P-glycoprotein. *Eur. J. Cancer*, **29A**, 408–415.

COLEY, H.M., TWENTYMAN, P.R. & WORKMAN, P. (1989*a*). Identification of anthracyclines and related agents that retain preferential activity over adriamycin in multidrug resistant cell lines and further resistance modification by verapamil and cyclosporin A. *Cancer Chemother. Pharmacol.*, **24**, 284–290.

COLEY, H.M., TWENTYMAN, P.R. & WORKMAN, P. (1989*b*). Improved cellular accumulation is characteristic of anthracyclines which retain high activity in multidrug resistant cell lines, alone or in combination with verapamil or cyclosporin A. *Biochem. Pharmacol.*, **38**, 4467–4475.

COLEY, H.M., TWENTYMAN, P.R. & WORKMAN, P. (1990). 9-Alkyl morpholinyl anthracyclines in the circumvention of multidrug resistance. *Eur. J. Cancer*, **26**, 665–667.

COLEY, H.M., WORKMAN, P. & TWENTYMAN, P.R. (1991). Retention of activity by selected anthracyclines in a multidrug resistant human large cell lung carcinoma line without P-glycoprotein hyperexpression. *Br. J. Cancer*, **63**, 351–357.

DE DUVE, C., DE BARSYI, T., POOLE, B., TROUET, A., TULKENS, P. & VAN HOOF, F. (1974). Lysosomotropic agents. *Biochem. Pharmacol.*, **23**, 2495–2551.

DIETEL, M. & SEIDEL, A. (1990). Morphologic alterations in drug sensitive *vs* drug resistant cells due to cytostatic application. *Cancer Treat. Rev.*, **17**, 3–10.

EGORIN, M.J., HILDEBRAND, R.C., CIMONO, E.F. & BACHUR, N.R. (1974). Cytofluorescence localisation of Adriamycin and Daunorubicin. *Cancer Res.*, **34**, 2243–2245.

EGORIN, M.J., CLAWSON, R.E., ROSS, L.A., SCHLOSSBERGER, N.M. & BACHUR, N.R. (1979). Cellular accumulation and disposition of aclacinomycin A. *Cancer Res.*, **39**, 4396–4676.

EGORIN, M.J., CLAWSON, R.E., COHEN, J.L., ROSS, L.A. & BACHUR, N.R. (1980). Cytofluorescence localisation of anthracycline antibiotics. *Cancer Res.*, **40**, 4669–4676.

GERVASONI, J.E., FIELDS, S.Z., KRISHNA, S., BAKER, M.A., ROSADO, M., THURAISAMY, K., HINDENBURG, A.A. & TAUB, R.N. (1991). Subcellular distribution of daunorubicin in P-glycoprotein-positive and -negative drug resistant cell lines using laser-assisted confocal microscopy. *Cancer Res.*, **51**, 4955–4963.

GIGLI, M., RASOANAIVO, T.W.D., MILLOT, J.-M., JEANNESSON, P., RIZZO, V., JARDILLIER, J.-C., ARCAMONE, F. & MANFAIT, M. (1989). Correlation between growth inhibition and intranuclear doxorubicin and 4′-deoxy′-iodo-doxorubicin quantitated in living K562 cells by microspectrofluorimetry. *Cancer Res.*, **49**, 560–564.

HILL, B.T., DENNIS, L.Y., LI, X.T. & WHELAN, R.D.H. (1985). Identification of anthracycline analogues with enhanced cytoxicity and lack of cross-resistance to adriamycin using a series of mammalian cell lines in vitro. *Cancer Chemother. Pharmacol.*, **14**, 194–201.

HINDENBURG, A.A., GERVASONI, J.E., KRISHNA, S., STEWART, V.J., ROSADO, M., LUTZKY, J., BHALLA, K., BAKER, M.A. & TAUB, R.N. (1989). Intracellular distribution and pharmacokinetics of daunorubicin in anthracycline-sensitive and -resistant HL-60 cells. *Cancer Res.*, **49**, 4607–4614.

KEIZER, H.G., SCHUURHUIS, G.J., BROXTERMAN, H.J., LANKELMA, J., SCHOONEN, W.G.E.J., VAN RIJN, J., PINEDO, H.M. & JOENJE, H. (1989). Correlation of multidrug resistance with decreased drug accumulation altered subcellular drug distribution and increased P-glycoprotein expression in cultured SW-1573 human lung tumour cells. *Cancer Res.*, **49**, 2988–2993.

KRISHAN, A., ISRAEL, M., MODEST, E.J. & FREI, E. (1976). Differences in cellular uptake and cytofluorescence of adriamycin and N-trifluoroacetyl adriamycin-14-valerate. *Cancer Res.*, **36**, 2108–2109.

LELONG, I.H., GUZIKOWSKI, A.P., HAUGLAND, R.P., PASTAN, I., GOTTESMAN, M.M. & WILLINGHAM, M. (1991). Fluorescent verapamil derivative for monitoring activity of multidrug transporter. *Mol. Pharmacol.*, **40**, 490–494.

LOTHSTEIN, L., SWEATMAN, T.W., DOCKTER, M.E. & ISRAEL, M. (1992). Resistance to N-benzyladriamycin-14-valerate in mouse J774.2 cells: P-glycoprotein expression without reduced N-benzyladriamycin-14-valerate accumulation. *Cancer Res.*, **52**, 3409–3417.

MARQUARDT, D. & CENTER, M.S. (1992). Drug transport mechanisms in HL60 cells isolated for resistance to adriamycin: evidence for nuclear drug accumulation and redistribution in resistant cells. *Cancer Res.*, **52**, 3157–3163.

MCGRATH, T. & CENTER, M.S. (1988). Mechanisms of multidrug resistance in HL-60 cells: evidence that a surface membrane protein distinct from P-glycoprotein contributes to reduced cellular accumulation of drug. *Cancer Res.*, **48**, 3559–3563.

REEVE, J.G., RABBITTS, P.H. & TWENTYMAN, P.R. (1990). Non-P glycoprotein mediated multidrug resistance with reduced EGF receptor expression in a human large cell lung cancer cell line. *Br. J. Cancer*, **61**, 851–855.

ROSS, K.L., STOLINSKY, D.C. & TOKES, Z.A. (1986). Accumulation of adriamycin into cytoplasmic compartments by resistant L-1210 cells. *Proc. Am. Assoc. Cancer Res.*, **27**, 273.

SCHUURHUIS, G.J., BROXTERMAN, H.J., CERVANTES, A., VAN HEININGEN, T.H.M., DE LANGE, J.H.M., BAAK, J.P.A., PINEDO, H.M. & LANKELMA, J. (1989). Quantitative determination of factors contributing to doxorubicin resistance in multidrug-resistant cells. *J. Natl Cancer Inst.*, **81**, 1887–1892.

SCHUURHUIS, G.J., BROXTERMAN, J.H., DE LANGE, J.H.M., PINEDO, H.M., VAN HEININGEN, T.H.M., KUIPER, C.M., SCHEFFER, G.L., SCHEPER, R.J., VAN KALKEN, C.K., BAAK, J.P.A. & LANKELMA, J. (1991). Early multidrug resistance, defined by changes in intracellular doxorubicin distribution, independent of P-glycoprotein. *Br. J. Cancer*, **64**, 857–861.

SCOTT, C.A., WESTMACOTT, D., BROADHURST, M.J., THOMAS, G.J. & HALL, M.J. (1986). 9-Alkyl anthracyclines. Absence of cross-resistance to adriamycin in human and murine cell cultures. *Br. J. Cancer*, **53**, 595–600.

SHOJI, Y., FISHER, M.H., PERIASAMY, A., HERMAN, B. & JULIANO, R.L. (1991). Verapamil and cyclosporin A modulate doxorubicin toxicity by distinct mechanisms. *Cancer Lett.*, **57**, 209–218.

STREETER, D.G., TAYLOR, D.L., ACTON, E.M. & PETERS, J.H. (1985). Comparative activities of various morpholinyl anthracyclines. *Cancer Chemother. Pharmacol.*, **14**, 160–164.

TSURUO, T., IIDA, H., KITATANI, Y., YOKOTA, K., TSUKAGOSHI, S. & SAKURAI, Y. (1984). Effect of quinidine and related compounds on cytotoxicity and cellular accumulation of vincristine and Adriamycin in drug-resistant tumour cells. *Cancer Res.*, **44**, 4303–4307.

TWENTYMAN, P.R., FOX, N.E., WRIGHT, K.A., WORKMAN, P., BROADHURST, M.J., MARTIN, J.A. & BLEEHEN, N.M. (1986). The *in vitro* effects and cross-resistance patterns of some novel anthracyclines. *Br. J. Cancer*, **53**, 585–594.

TWENTYMAN, P.R., WRIGHT, K.A., REEVE, J.G. & KOCH, G. (1990). Chemosensitisation by verapamil and cyclosporin A in mouse tumour cells expressing different levels of P-glycoprotein and CP22 (sorcin). *Br. J. Cancer*, **62**, 89–93.

WHITE, J.G., AMOS, W.B. & FORDHAM, M. (1987). An evaluation of confocal *vs* conventional imaging of biological structures by fluorescence light microscopy. *J. Cell Biol.*, **105**, 41–48.

WILLINGHAM, M., CORNWELL, M.M., CARARELLI, C.O., GOTTESMAN, M.M. & PASTAN, I. (1986). Single cell analysis of daunomycin uptake and efflux in multidrug-resistant and sensitive KB cells: effects of verapamil and other drugs. *Cancer Res.*, **46**, 5941–5946.

ZAMORA, J.M. & BECK, W.T. (1986). Potentiation of vinca alkaloid cytotoxicity by aminolines, acridines, indole alkaloids and aromatic amines. *Proc. Am. Assoc. Cancer Res.*, **27**, 395.